# Supplementary material for: Investigating disagreement in the scientific literature
Source: eLife. 2021 Dec 24;10:e72737. doi: 10.7554/eLife.72737 (PMC8709576; doi:10.7554/eLife.72737)
Supplement: Supplementary file 1. [file elife-72737-supp1.docx]

**Investigating disagreement in the scientific literature**

**(Lamers *et al*. 2021 *eLife* 10:e72737)**

**Supplementary file 1**

*Tables S1–S4*

**Table S1**

|  | **_standalone_** | **+studies** | **+ideas** | **+methods** | **+results** |
| --- | --- | --- | --- | --- | --- |
| ***challenge**** | 405,613 | 16,120 | 7,114 | 13,806 | 15,352 |
| ***conflict**** | 212,246 | 22,190 | 3,603 | 5,560 | 49,961 |
| ***contradict**** | 115,375 | 19,509 | 5,482 | 2,793 | 52,648 |
| ***contrary*** | 171,711 | 17,207 | 3,651 | 4,699 | 27,273 |
| ***contrast**** | 1,257,866 | 116,450 | 7,774 | 37,372 | 119,181 |
| ***controvers**** | 154,608 | 12,187 | 1,840 | 3,028 | 15,473 |
| ***debat**** | 150,617 | 8,509 | 1,774 | 2,678 | 4,663 |
| ***differ**** | 2,003,677 | 100,764 | 9,531 | 85,309 | 110,599 |
| ***disagree**** | 52,615 | 5,724 | 1,142 | 1,682 | 12,459 |
| ***disprov**** | 2,938 | 278 | 528 | 100 | 358 |
| ***no consensus*** | 16,632 | 1,424 | 37 | 830 | 421 |
| ***questionable*** | 24,244 | 1,045 | 852 | 1,175 | 2,050 |
| ***refut**** | 10,322 | 1,399 | 1,564 | 338 | 2,262 |

Number of citances in the Elsevier ScienceDirect database containing signal term (rows) and filter term (columns) combination. For some signal terms, variants are excluded; for example, “not contradict” is not matched. For filter terms, “_standalone_” indicates that only the signal term was used to query. The remaining columns, +studies, +ideas, +methods, and +results correspond to sets of filter terms outlined in Table S2.

**Table S2**

| **Filter term** | **Valid** | **Example** |
| --- | --- | --- |
| **_standalone_** | Yes | Although phosphorus has traditionally been seen as the limiting nutrient in freshwater ecosystems […], more recent evidence has begun to **challenge** this view and has demonstrated that both nitrogen and phosphorus can limit, or at least co-limit, primary production in freshwaters […]. |
| **_standalone_** | No | Analogs of these molecules have shown up to 1000-fold higher activity but are a great **challenge** to delivery because of their extreme hydrophobicity […]. |
| **+studies** | Yes | However, recent studies have **challenged** this survival benefit in comparison with current usual care […]. |
| **+studies** | No | The low affinity with which volatile general anesthetics bind to macromolecules has made conclusive identification of the in vivo targets by direct binding studies a **challenge** […]. |
| **+ideas** | Yes | This result **challenges** AUM theory […] and some prior research […]. |
| **+ideas** | No | The description of the resonant electron capture by molecules connected with the formation of negative ions represents still the **challenge** for the **theory** […]. |
| **+methods** | Yes | This model has since been **challenged** by claims that Helderberg formation boundaries are isochronous across the basin […]. |
| **+methods** | No | Subsequent studies in the human **challenge** model have also supported the role of NA-specific antibody in protection […]. |
| **+results** | Yes | It has been reported that the prevalence of autoimmune disorders in celiac disease is related to the duration of exposure to gluten […], although this result has been **challenged** […]. |
| **+results** | No | Some of the larger **challenges** identified in Africa include data collection, access and management, infrastructure and capacity […]. |

Examples of valid and invalid citances returned for "challenge*" and filter term combinations. Signal terms are bolded and underlined, whereas relevant filter terms are underlined but not bolded. “[…]” has been used in places of reference names or numbers.

**Table S3**

| Label | Signal | Filter | Valid | Example |
| --- | --- | --- | --- | --- |
| **I** | conflict* | None | No | “For instance, […] study **conflicts** based on ethnicity where ethnic identity works as a device to enforce coalition membership.” |
| **II** | disprove* | None | No | “These techniques are typically used to confirm or **disprove** an a priori hypothesized model, i.e. to test the statistical adequacy of a proposed causal model […]” |
| **III** | challenge* | +ideas | Yes | “Reversal theory **challenges** the idea of personality traits in suggesting that people fluctuate between metamotivational states that are opposite and mutually exclusive […]” |
| **IV** | contradict* | +methods | Yes | “The existence of topological singularities is in **contradiction** with […]’s method of continuous transformations of a rectangular Cartesian frame of coordinates into a curvilinear grid without singularities […].” |
| **V** | controvers* | +ideas | Yes | “There is still a considerable **controversy** about the idea of homology between specific areas of rat and primate PFC […].” |
| **VI** | disagree* | +results | Yes | “These results were in **disagreement** with much of the literature, where there is consensus that, H2 is almost exclusively catalyzed by SRB at low COD/SO42- ratios […]” |
| **VII** | no consensus | +ideas | Yes | “Because of the controversial data collected, **no consensus** about this theory has been reached to date […].” |
| **VIII** | contradict* | +methods | Yes | “Thus, the approach is somewhat **contradictory** to certain approaches in robotics that strive to develop highly autonomous robots capable of performing independent decisions based on sensory data […].” |

Examples of citances from notable signal/filter phrase combinations labeled in Figure XYZ. “[…]” has been used in places of reference names or numbers. Relevant signal terms have been bolded and underlined, whereas relevant filter terms have only been underlined.

**Table S4**

| **Quantity** | **Validity cutoff** | **All**  **Fields** | **Soc & Hum** | **Bio & Health** | **Life & Earth** | **Phys & Engr** | **Math & Comp** |
| --- | --- | --- | --- | --- | --- | --- | --- |
| **Overall** | 80% | 0.32% | 0.61% | 0.41% | 0.29% | 0.15% | 0.06% |
|  | 70% | 0.40% | 0.78% | 0.50% | 0.36% | 0.20% | 0.15% |
| **Change per year** | 80% | -0.0005 | -0.0033 | +0.0017 | +0.0018 | -0.0045 | -0.0019 |
|  | 70% | -0.0005 | -0.0042 | +0.0022 | +0.0019 | -0.0061 | -0.0028 |

Results are robust to both the 80% and 70% validity cutoffs. Quantities of interest using the 23 queries above the 80% validity cutoff, and the 36 queries above the 70% validity cutoff. Shown are the overall rates of disagreement and the change in the share of disagreement per year.
