# Supplementary material for: Investigating disagreement in the scientific literature
Source: eLife. 2021 Dec 24;10:e72737. doi: 10.7554/eLife.72737 (PMC8709576; doi:10.7554/eLife.72737)
Supplement: Supplementary file 2. [file elife-72737-supp2.docx]

**Investigating disagreement in the scientific literature**

**(Lamers *et al*. 2021 *eLife* 10:e72737)**

**Supplementary file 2**

***Papers with most disagreement citances and papers most often cited in the context of disagreement***

## Papers with most disagreement citances

While the extensive manual validation of our queries and results ensures the robustness of our analysis at an aggregate scale, the list of publications issuing most disagreement citations does reveal that it remains difficult to separate research object from commentary on cited material. Table S5 shows the papers that issue the most disagreement citations; going through these papers reveals several artifacts. “Debating” and “debates” are, for example, the object of study in Alén et al. (2015) and Doody & Condon (2012), and the citances in the paper reflect this, e.g. “students also seem to both enjoy debates and recognise their value” and “debate is effective in helping students learn a discipline and demonstrate the ability to read and write critically.” Controversy, likewise, is the subject in Ersoy (2010), Nam (2014), and Colston & Vadjunec (2015), as are environmental conflicts in Stepanova & Bruckmeier (2013). Bruschke & Divine (2017) makes frequent mention of the first televised US presidential *debate*. This leaves French & Koeberl (2010), Kalter (2003) and Millan (2006), three publications that do not immediately appear focused on subjects that would trigger our queries.

French & Koeberl (2010) discusses methods for identifying meteorite impact structures on earth, “as well as an overview of doubtful criteria or ambiguous lines of evidence that have erroneously been applied in the past”, and this paper indeed cites many sources in the context of controversy, e.g. “the identification of such glasses as impact or non-impact products is difficult and commonly controversial,” “the impact origin for many glasses still remains controversial and unconfirmed,” “there are also debates about the formation of maskelynite itself” and “the nature, characteristics, and causes of these changes have been widely studied and are still being debated.”

Kalter (2003), while classified as a full-length article, is a book that was also included in a special issue of the journal Neurotoxicology and Teratology. Considered a pillar in the study of congenital abnormalities, its exceptional length may account in part for its high number of disagreement citances. One of these citances describes “a discussion—debate better characterizes it—that took place in 1953,” but many others indeed refer to scientific disagreements within the field of study, e.g. “a Mayo Clinic study seemed to agree, despite conflicting evidence,” “a contrary finding came from Scotland, another high-risk region,” “an early analysis, as well as a later one, disagreed” and “earlier retrospective and prospective studies had been contradictory.”

Millan (2006) is a review article making a case for multi-target agents to treat depressive states. It likewise introduces a number of citances that indeed signify disagreement in the scientific literature, e.g. “the gravity of cognitive impairment in young patients is still debated,” “this notion remains somewhat controversial,” “its precise degree of efficacy in this regard is still debated” and “for recent critical discussions of these controversial issues–from a variety of viewpoints–see […].” However, a few false positives also occur, when citing the work of an author by the name of DeBattisa, whose name was caught by our *debate* query.

**Table S5. Papers that introduced the most disagreement citances**

| Total citances | Disagreement citances | Publication | Title | Document type |
| --- | --- | --- | --- | --- |
| 50 | 27 | Alén, Domínguez, & De Carlos (2015) | University students' perceptions of the use of academic debates as a teaching methodology | Full-length Article |
| 400 | 27 | French & Koeberl (2010) | The convincing identification of terrestrial meteorite impact structures: What works, what doesn't, and why | Review Article |
| 66 | 26 | Doody & Condon (2012) | Increasing student involvement and learning through using debate as an assessment | Full-length Article |
| 64 | 25 | Ersoy (2010) | Social studies teacher candidates' views on the controversial issues incorporated into their courses in Turkey | Full-length Article |
| 91 | 24 | Nam (2014) | The effects of trust and constructive controversy on student achievement and attitude in online cooperative learning environments | Full-length Article |
| 1292 | 23 | Kalter (2003) | Teratology in the 20th century Environmental causes of congenital malformations in humans and how they were established | Full-length Article |
| 1708 | 23 | Millan (2006) | Multi-target strategies for the improved treatment of depressive states: Conceptual foundations and neuronal substrates, drug discovery and therapeutic application | Review Article |
| 50 | 21 | Bruschke & Divine (2017) | Debunking Nixon’s radio victory in the 1960 election: Re-analyzing the historical record and considering currently unexamined polling data | Full-length Article |
| 107 | 21 | Stepanova & Bruckmeier (2013) | The relevance of environmental conflict research for coastal management. A review of concepts, approaches and methods with a focus on Europe | Review Article |
| 74 | 20 | Colston & Vadjunec (2015) | A critical political ecology of consensus: On “Teaching Both Sides” of climate change controversies | Full-length Article |

## Papers most often cited in the context of disagreement

We also examine disagreement form the cited paper perspective, that is, by looking at those papers that received the most paper-level disagreement citations and which were most often cited in the context of community-level disagreement. Table S6 lists these papers, and again we reveal issues with methodological artifacts, but also highlight interesting instances of controversy in the literature. The majority of these publications relate to plate tectonics, and in particular, the North China Craton. These papers include Zhao, Sun, Wilde, and Sanzhong (2005), Kusky and Li (2003), Zhao, Wilde, Cawood, and Sun (2001), Zhai and Santosh (2011)¸ Wilde, Zhao, and Sun (2002) and Kusky (2011). Li et al. (2008) also appears to be closely related. Several of these publications have authors in common, notably Zhao (3), Wilde (3), Sun (3), Kusky (2) and Li (2). Zhai and Santosh (2011) summarizes the situation as follows:

“A long controversy and debate surround the evolution of the NCC, particularly the timing and tectonic processes involved in the amalgamation of the Eastern (Yanliao) and Western (Ordos and Yinshan) Blocks along the Central Orogenic Belt. One school of thought proposes an east-directed subduction of an old ocean, with final collision between the two blocks at ~ 1.85 Ga <several citations to Zhao’s work>. In contrast, some others suggest a westward subduction, with final collision between the two blocks to form the NCC at ~ 2.5 Ga <several citations to Kusky’s work>.”

The majority of disagreement citances to these papers also mention this long-standing scientific controversy, including phrases such as,

- “the number of continental blocks and the mechanism by which they were welded together to form the coherent basement remain controversial,”
- “tectonic history of this central region is in debate,”
- “it is currently debated how the collisional processes proceeded,”
- “controversy still remains as to the timing and tectonic processes involved,”
- “it still remains controversial as to how the craton should be subdivided and where the collisional boundaries are located,”
- “models that evaluate the Paleoproterozoic crustal evolution of the NCC remain controversial,”
- “the timing of the collision between these blocks remains controversial,”
- “this controversy is also reflected in various tectonic models for the NCC'' and
- “the time of the amalgamation between the Eastern Block and the Western block is still debated,”

These phrases are often accompanied by a large number of citations to papers by Zhao and Kusky, with both authors either explicitly posed as opposing one another like in the example from Zhai & Santosh (2011) above, or citations to their papers grouped together as if to present a large body of conflicting literature. It is clear that this scientific controversy dominates scholarship on the North China Craton, to the point that even Wikipedia mentions it extensively (Wikipedia, 2021). It should be noted that the works by Zhao and Kusky also receive a fair share of citations that mention their empirical and theoretical contributions, without the context of disagreement, making material research contributions to both their preferred model and the research on the craton at large. The high share of disagreement citances to these papers appears to stem from the highly divided nature of their research field.

**Table S6. Publications that were most cited in the context of disagreement.**

| Total citances | Disagreement citances | Publication | Title | Document type |
| --- | --- | --- | --- | --- |
| 389 | 99 | Munro (2003) | Lipid Rafts Elusive or Illusive? | Review Article |
| 477 | 99 | Murphy et al. (2009) | The impact of global signal regression on resting state correlations: Are anti-correlated networks introduced? | Full-length Article |
| 1753 | 83 | Zhao, Sun, Wilde, & Sanzhong (2005) | Late Archean to Paleoproterozoic evolution of the North China Craton: key issues revisited | Full-length Article |
| 1344 | 69 | Li et al. (2008) | Assembly, configuration, and break-up history of Rodinia: A synthesis | Full-length Article |
| 663 | 65 | Kusky & Li (2003) | Paleoproterozoic tectonic evolution of the North China Craton | Full-length Article |
| 1377 | 64 | Zhao, Wilde, Cawood, & Sun (2001) | Archean blocks and their boundaries in the North China Craton: lithological, geochemical, structural and P–T path constraints and tectonic evolution | Full-length Article |
| 972 | 51 | Zhai & Santosh (2011) | The early Precambrian odyssey of the North China Craton: A synoptic overview | Full-length Article |
| 50 | 45 | Debat et al. (2003) | A new metamorphic constraint for the Eburnean orogeny from Paleoproterozoic formations of the Man shield (Aribinda and Tampelga countries, Burkina Faso) | Full-length Article |
| 448 | 45 | Wilde, Zhao, & Sun (2002) | Development of the North China Craton During the Late Archaean and its Final Amalgamation at 1.8 Ga: Some Speculations on its Position Within a Global Palaeoproterozoic Supercontinent | Full-length Article |
| 247 | 43 | Kusky (2011) | Geophysical and geological tests of tectonic models of the North China Craton | Full-length Article |

The three remaining papers cover different topics. Munro (2003) reviews literature on lipids in cell membranes of eukaryotic cells and discusses the (non)existence of lipid rafts. It points out that, regardless of enthusiasm for the lipid raft model in the research community, observations of these raft structures are problematic and several factors exist that cast doubt on the model. The existence of lipid rafts should therefore be treated as hypothetical rather than established fact. Citances that mention this paper in the context of disagreement also appear to use it as an exemplar of controversy in the field, with phrasing such as,

- “the entity of lipid microdomains is controversial,”
- “the existence of noncaveolar lipid rafts in vivo is still debated,”
- “this early operative definition of lipid rafts was subject of much debate,”
- “the lipid raft concept has been controversial since it was introduced several decades ago” and
- “a number of points have been recently debated in the literature.”

Even many citances that do not qualify as disagreement per our operationalization appear to still signal it, e.g.

- “rafts still remain a hypothesis,”
- “evidence that lipid rafts exist in living cells remains elusive,”
- “the classical perception of rafts as stable entities within the fluid bilayer has provoked some opposition,”
- “the biological substrate for this notion is not clearly defined” and
- “isolation of DRM or lipid rafts is however a delicate matter.”

This paper, specifically, serves as a focal point of controversy within the community researching lipid rafts precisely because its primary purpose appears to be to create this controversy; raising concerns about the lipid raft model and calling for a reevaluation of its canonicity in the face of shaky foundational evidence of the existence of these rafts. This is different from the North China Craton papers, where controversy is long established in the field.

Murphy et al. (2009) focuses on a pre-processing method used in low-frequency fMRI research, called global signal regression. This paper alleges that the method is inadequate and “may cause spurious findings of negatively correlated regions in the brain.” This paper appears to have heralded a change in how data is handled in this research field, with many citances mentioning it to explain why data was processed differently, e.g.

- “the global signal was not regressed out due to its controversial biological interpretations,”
- “given the controversy of removing the global signal in the preprocessed rs-fMRI data, we did not regress the global signal out in the present study,”
- “global signal regression is a somewhat controversial part of the preprocessing pipeline for resting state MRI data, and was not performed in this study” and
- “given the controversy of removing the global signal in the preprocessing of R-fMRI data […], we did not regress the global signal out.”

Other citances in the context of disagreement simply point out this controversy, e.g.

- “there is ongoing debate as to the nature of anti-correlations introduced by global signal regression”
- “in recent years there has been an ongoing debate on global signal removal in the preprocessing.”

As with the lipid raft paper, many citances that do not qualify as disagreement also embrace the controversy, as evident in phrasing such as “global-signal was not included in the model for its effects on brain–behavior correlations” and “we decided against this approach as several recent studies showed that global signal regression may significantly bias connectivity analyses.”

Finally, Debat et al. (2003) is another example of a false positive result of our approach, in which the lead author’s name activated the *debate* query. While this is unfortunate, our extensive manual validation of our query results shows that despite this prominent false positive, there were no large systemic flaws in our approach that might otherwise color our analysis at the aggregate level. While eliminating cited author names from citances at scale is not trivial, this example serves to stress the importance of text pre-processing.

**References**

Alén, E., Domínguez, T., & de Carlos, P. (2015). University students׳ perceptions of the use of academic debates as a teaching methodology. *Journal of Hospitality, Leisure, Sport & Tourism Education*, **16:**15–21. https://doi.org/10.1016/j.jhlste.2014.11.001

Bruschke, J., & Divine, L. (2017). Debunking Nixon’s radio victory in the 1960 election: Re-analyzing the historical record and considering currently unexamined polling data. *The Social Science Journal*, **54:**67–75. https://doi.org/10.1016/j.soscij.2016.09.007

Colston, N. M., & Vadjunec, J. M. (2015). A critical political ecology of consensus: On “Teaching Both Sides” of climate change controversies. *Geoforum*, **65:**255–265. https://doi.org/10.1016/j.geoforum.2015.08.006

Debat, P., Nikiéma, S., Mercier, A., Lompo, M., Béziat, D., Bourges, F., Roddaz, M., Salvi, S., Tollon, F., & Wenmenga, U. (2003). A new metamorphic constraint for the Eburnean orogeny from Paleoproterozoic formations of the Man shield (Aribinda and Tampelga countries, Burkina Faso). *Precambrian Research*, **123:**47–65. <https://doi.org/10.1016/S0301-9268(03)00046-9>

Doody, O., & Condon, M. (2012). Increasing student involvement and learning through using debate as an assessment. *Nurse Education in Practice*, **12:**232–237. https://doi.org/10.1016/j.nepr.2012.03.002

Ersoy, A. F. (2010). Social studies teacher candidates’ views on the controversial issues incorporated into their courses in Turkey. *Teaching and Teacher Education*, **26:**323–334.

French, B. M., & Koeberl, C. (2010). The convincing identification of terrestrial meteorite impact structures: What works, what doesn’t, and why. *Earth-Science Reviews*, **98:**123–170. https://doi.org/10.1016/j.earscirev.2009.10.009

Kalter, H. (2003). Teratology in the 20th century: Environmental causes of congenital malformations in humans and how they were established. *Neurotoxicology and Teratology*, **25:**131–282. https://doi.org/10.1016/S0892-0362(03)00010-2

Kusky, T. M. (2011). Geophysical and geological tests of tectonic models of the North China Craton. *Gondwana Research*, **20:**26–35. <https://doi.org/10.1016/j.gr.2011.01.004>

Kusky, T. M., & Li, J. (2003). Paleoproterozoic tectonic evolution of the North China Craton. *Journal of Asian Earth Sciences*, **22:**383–397. <https://doi.org/10.1016/S1367-9120(03)00071-3>

Li, Z. X., Bogdanova, S. V., Collins, A. S., Davidson, A., De Waele, B., Ernst, R. E., Fitzsimons, I. C. W., Fuck, R. A., Gladkochub, D. P., Jacobs, J., Karlstrom, K. E., Lu, S., Natapov, L. M., Pease, V., Pisarevsky, S. A., Thrane, K., & Vernikovsky, V. (2008). Assembly, configuration, and break-up history of Rodinia: A synthesis. *Precambrian Research*, **160:**179–210. https://doi.org/10.1016/j.precamres.2007.04.021

Millan, M. J. (2006). Multi-target strategies for the improved treatment of depressive states: Conceptual foundations and neuronal substrates, drug discovery and therapeutic application. *Pharmacology & Therapeutics*, **110:**135–370. https://doi.org/10.1016/j.pharmthera.2005.11.006

Munro, S. (2003). Lipid Rafts: Elusive or Illusive? *Cell*, **115:**377–388. https://doi.org/10.1016/S0092-8674(03)00882-1

Murphy, K., Birn, R. M., Handwerker, D. A., Jones, T. B., & Bandettini, P. A. (2009). The impact of global signal regression on resting state correlations: Are anti-correlated networks introduced? *NeuroImage*, **44:**893–905. https://doi.org/10.1016/j.neuroimage.2008.09.036

Nam, C. W. (2014). The effects of trust and constructive controversy on student achievement and attitude in online cooperative learning environments. *Computers in Human Behavior*, **37:**237–248. https://doi.org/10.1016/j.chb.2014.05.007

Stepanova, O., & Bruckmeier, K. (2013). The relevance of environmental conflict research for coastal management. A review of concepts, approaches and methods with a focus on Europe. *Ocean & Coastal Management*, **75:**20–32. https://doi.org/10.1016/j.ocecoaman.2013.01.007

Wilde, S. A., Zhao, G., & Sun, M. (2002). Development of the North China Craton During the Late Archaean and its Final Amalgamation at 1.8 Ga: Some Speculations on its Position Within a Global Palaeoproterozoic Supercontinent. *Gondwana Research*, **5:**85–94. https://doi.org/10.1016/S1342-937X(05)70892-3

Zhao, G., Sun, M., Wilde, S. A., & Sanzhong, L. (2005). Late Archean to Paleoproterozoic evolution of the North China Craton: Key issues revisited. *Precambrian Research*, **136:**177–202. <https://doi.org/10.1016/j.precamres.2004.10.002>

Zhao, G., Wilde, S. A., Cawood, P. A., & Sun, M. (2001). Archean blocks and their boundaries in the North China Craton: Lithological, geochemical, structural and P–T path constraints and tectonic evolution. *Precambrian Research*, **107:**45–73. https://doi.org/10.1016/S0301-9268(00)00154-

Zhai, M.-G., & Santosh, M. (2011). The early Precambrian odyssey of the North China Craton: A synoptic overview. *Gondwana Research*, **20:**6–25. <https://doi.org/10.1016/j.gr.2011.02.005>

Wikipedia. (2021). North China Craton. <https://en.wikipedia.org/w/index.php?title=North_China_Craton&oldid=1056978275> [Accessed: 13 December 2021]
